# A comparative study of irrigation techniques and the development of a self-serve training model for ophthalmology residents

**DOI:** 10.1186/s12909-025-06889-2

**Published:** 2025-03-06

**Authors:** Weifeng Huang, Zuohong Li, Yang Gao, Xiaohui Wang, Mingfeng Lu, Xianchai Lin, Xuanwei Liang, Rong Lu

**Affiliations:** State Key Laboratory of Ophthalmology, Zhongshan Ophthalmic Center, Sun Yat-sen University, Guangdong Provincial Key Laboratory of Ophthalmology and Visual Science, Guangdong Provincial Clinical Research Center for Ocular Diseases, Guangzhou, 510060 China

**Keywords:** Lacrimal irrigation, Training model, Haptic feedback, Self-serve

## Abstract

**Background:**

Lacrimal irrigation is a fundamental skill for diagnosing and managing lacrimal diseases. This study evaluates two lacrimal irrigation techniques and introduces a haptic-visual integrated self-serve training model to enhance skill acquisition among novice ophthalmology residents.

**Methods:**

Ninety-two ophthalmology residents were randomized into Group A (*n* = 47) and Group B (*n* = 45). Both groups completed an 8-hour training program comprising theoretical instruction, demonstrations, and hands-on practice. Group A provided feedback to refine the training model, which was subsequently implemented in Group B1 (*n* = 23), while Group B2 (*n* = 22) served as the control. Outcomes were assessed through skill evaluations and post-training questionnaires measuring confidence scores and perceived efficacy.

**Results:**

In Group A, 70.2% of participants preferred Technique 1 for its perceived ease of use, while 29.8% favored Technique 2 for pressurized irrigation scenarios (*p* < 0.05). Key barriers to proficiency included the absence of suitable training models (63.8%) and psychological anxiety (25.5%). In Group B, participants using the training model (Group B1) demonstrated significantly higher confidence scores compared to Group B2 (8.4 ± 1.2 vs. 6.1 ± 1.5, *p* < 0.05). Although skill assessment scores showed a positive trend in Group B1 (80.7 ± 8.3 vs. 76.8 ± 9.1), the difference was not statistically significant (*p* > 0.05).

**Conclusion:**

Both lacrimal irrigation techniques are equally accessible to novices, with Technique 2 offering advantages in pressurized irrigation. The self-serve training model significantly enhances procedural confidence and addresses critical training barriers, including resource limitations and psychological safety. Future studies should validate these findings in larger cohorts and refine the model to incorporate enhanced simulation techniques and dynamic physiological feedback.

**Supplementary Information:**

The online version contains supplementary material available at 10.1186/s12909-025-06889-2.

## Introduction

Lacrimal irrigation, a widely utilized and minimally invasive procedure, serves as a cornerstone in the clinical assessment of obstructive and inflammatory conditions affecting the lacrimal system [1, 2]. Its therapeutic potential was recognized as early as 1956, with reports indicating that forced irrigation successfully resolved nasolacrimal duct obstruction in 50% of patients [3]. In recent years, however, the primary application of lacrimal irrigation has shifted toward diagnostic purposes [4]. This procedure plays a pivotal role in identifying lacrimal pathologies by providing critical insights into the location and nature of obstructions within the lacrimal duct. Furthermore, lacrimal irrigation is an essential preoperative evaluation for intraocular surgeries, given that lacrimal obstruction is a well-documented risk factor for postoperative endophthalmitis [5, 6].

While advanced diagnostic modalities such as dacryocystography and dacryoscintigraphy offer superior accuracy in evaluating the anatomy and function of the lacrimal drainage system, their widespread adoption is hindered by limitations in time, resources, and accessibility, particularly in community healthcare settings [2, 7, 8]. These methods are unsuitable for routine screening of lacrimal drainage system diseases and are typically reserved for cases where abnormal findings are detected during lacrimal irrigation. Consequently, lacrimal irrigation remains an indispensable diagnostic tool in ophthalmology.

Given the clinical significance and accessibility of lacrimal irrigation, proficiency in this fundamental skill is essential for ophthalmology residents. However, many trainees frequently lack self-confidence during their initial attempts to master the procedure. This challenge is exacerbated by insufficient training opportunities and the absence of appropriate simulation models, resulting in a prolonged and inefficient learning process.

More critically, the direct training of residents on patients raises substantial ethical and legal concerns. The inexperience of trainees increases the risk of iatrogenic injuries, as exemplified by a reported case in which a detached hypodermic needle during ocular irrigation led to corneal injury, iris perforation, and severe endophthalmitis [9]. Additionally, issues related to informed consent arise, as patients may not be fully aware that they are being treated by a trainee, potentially leading to ethical dilemmas and legal complications. These challenges underscore the urgent need for alternative training methods that minimize patient involvement while ensuring effective skill acquisition. An appropriate simulation model could provide a safe and controlled environment for residents to practice lacrimal irrigation techniques, thereby reducing unnecessary risks to patients.

Moreover, the technical proficiency of the practitioner significantly influences the reliability of lacrimal irrigation outcomes. Variations in technique can lead to inconsistent results, particularly in the assessment of reflux patterns, which serve as crucial diagnostic indicators. Current research presents divergent benchmarks for normal reflux, with some studies suggesting that patent lacrimal systems exhibit less than 20% reflux [10, 11], while others set the threshold at 0% [12–14]. Although the interpretation of reflux remains somewhat subjective, it is essential to minimize technique-related variations to ensure diagnostic accuracy and reliability in clinical assessments.

To address these challenges, efficient training methodologies and accessible, effective training models are imperative. However, research on lacrimal irrigation training for ophthalmology residents remains limited, particularly in comparing techniques for skill acquisition and retention, and in developing standardized models that simulate clinical scenarios while minimizing patient risk.

This study introduces two distinct techniques and proposes a haptic-visual integrated self-serve training model. The acceptability and efficacy of these approaches are systematically evaluated to enhance lacrimal irrigation proficiency among residents.

## Methods

### Participants and post-training questionnaire survey

A total of 92 ophthalmology residents, currently in the pre-clinical stage of their residency training, participated in this study. All participants were novice learners in ophthalmic basic skills with limited clinical experience and were divided into two groups: Group A (47 participants) and Group B (45 participants).

Group A underwent an 8-hour training session where they were instructed in two different techniques for lacrimal irrigation by a senior instructor. During the training session, the participants practice lacrimal irrigation with each other under the guidance of the senior instructor. Skill assessments were conducted for all participants after the training session. In addition, post-training questionnaires were also used to assess their abilities and gather feedback (Supplemental Table 1). Data were collected on their baseline skills, practice times, and proficiency levels (Elementary, Intermediate, and Advanced). The operational difficulty, stability, and injection performance of each trainee’s chosen technique was also assessed. Furthermore, the operator assessed the subjects’ level of comfort by taking into account patient complaints and facial expressions. A numeric rating scale ranging from 0 to 10 was utilized, where 0 indicated no pain and 10 indicated severe pain.

Based on the feedback received from Group A, we developed a simplified training model. Subsequently, Group B also planned to receive the same training and was randomly divided into two subgroups using block randomization to ensure balanced group sizes and minimize selection bias: Group B1 (23 participants), which implemented the training model, and Group B2 (22 participants), which did not. Participants in Group B1 were guided to practice lacrimal irrigation with the assistance of the training model before proceeding to practice with their partners. Skill assessments were conducted for all participants in Group B after the training session. Additionally, post-training questionnaires (Supplemental Table 1) were used to evaluate the effectiveness of the training model in improving the mastery of lacrimal irrigation skills.

**Fig. 1:**
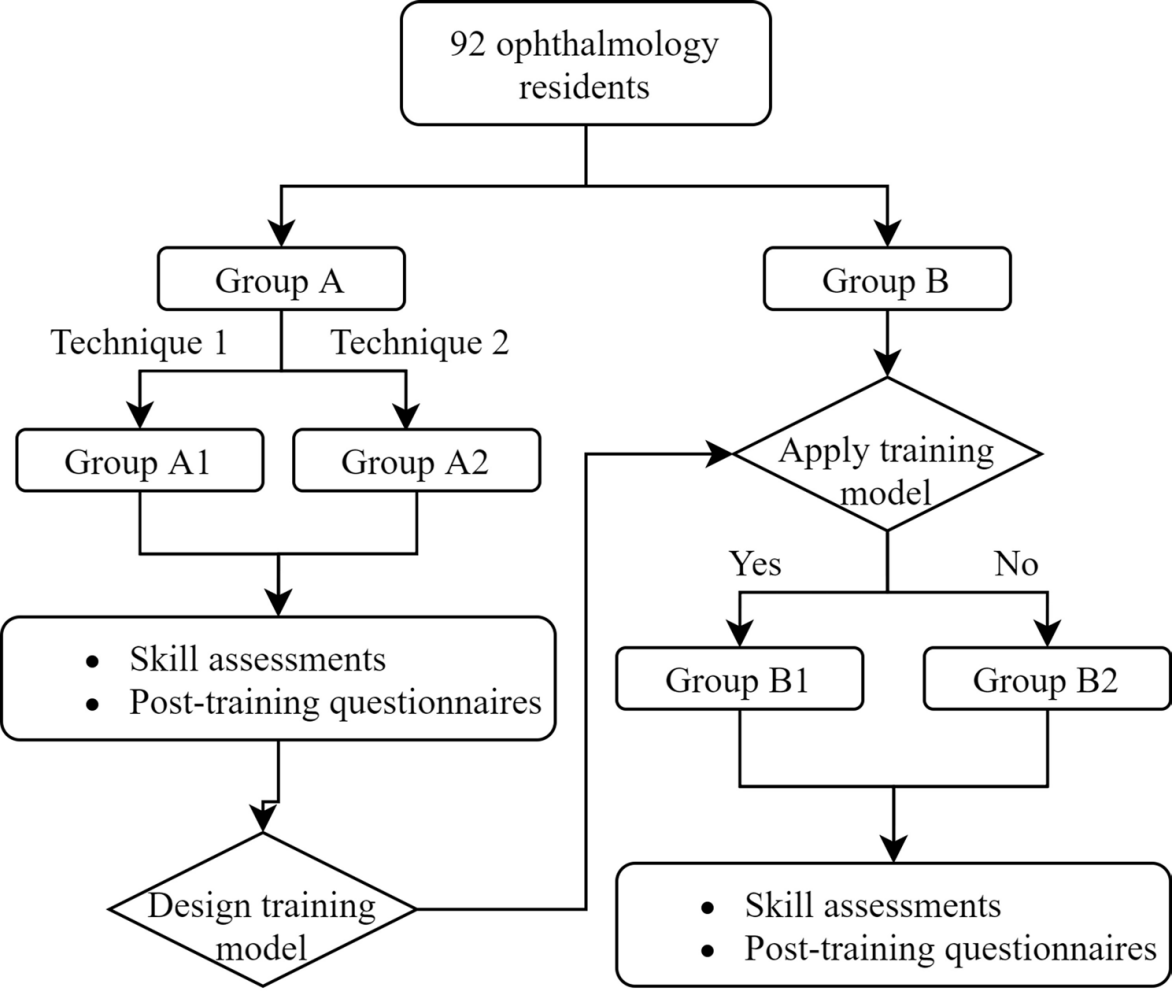
Flow chart of the study

In the analysis of Group B, participants with pre-existing entry-level skill ability were excluded. Entry-level skill ability was defined as the ability to independently and successfully perform lacrimal irrigation with a history of ≤ 10 prior attempts. This threshold was established based on the study team’s clinical experience and the assumption that participants with more than 10 successful attempts likely possess a level of proficiency beyond that of a novice learner. Although no specific guidelines or literature directly support this threshold, it was deemed appropriate to ensure a homogeneous novice cohort for evaluating the training model’s effectiveness (Fig. 1).

### Lacrimal irrigation test

#### Preparation of medicines and supplies

A 5mL syringe, blunt-tipped 23-G cannula, 0.9% saline solution, proparacaine hydrochloride eye drops, cotton swab, and punctum dilator were assembled.

#### Lacrimal irrigation process

Prior to the examination, participants with contraindications were excluded. Topical ocular surface anesthesia was administered using proparacaine hydrochloride eye drops. In cases where the punctum was stenosis, a punctum dilator was utilized.

A 23-G lacrimal cannula was attached to a 5 ml syringe containing a saline solution with a small amount of ointment. The operator positioned themselves behind the patient to begin lacrimal irrigation.

**Fig. 2:**
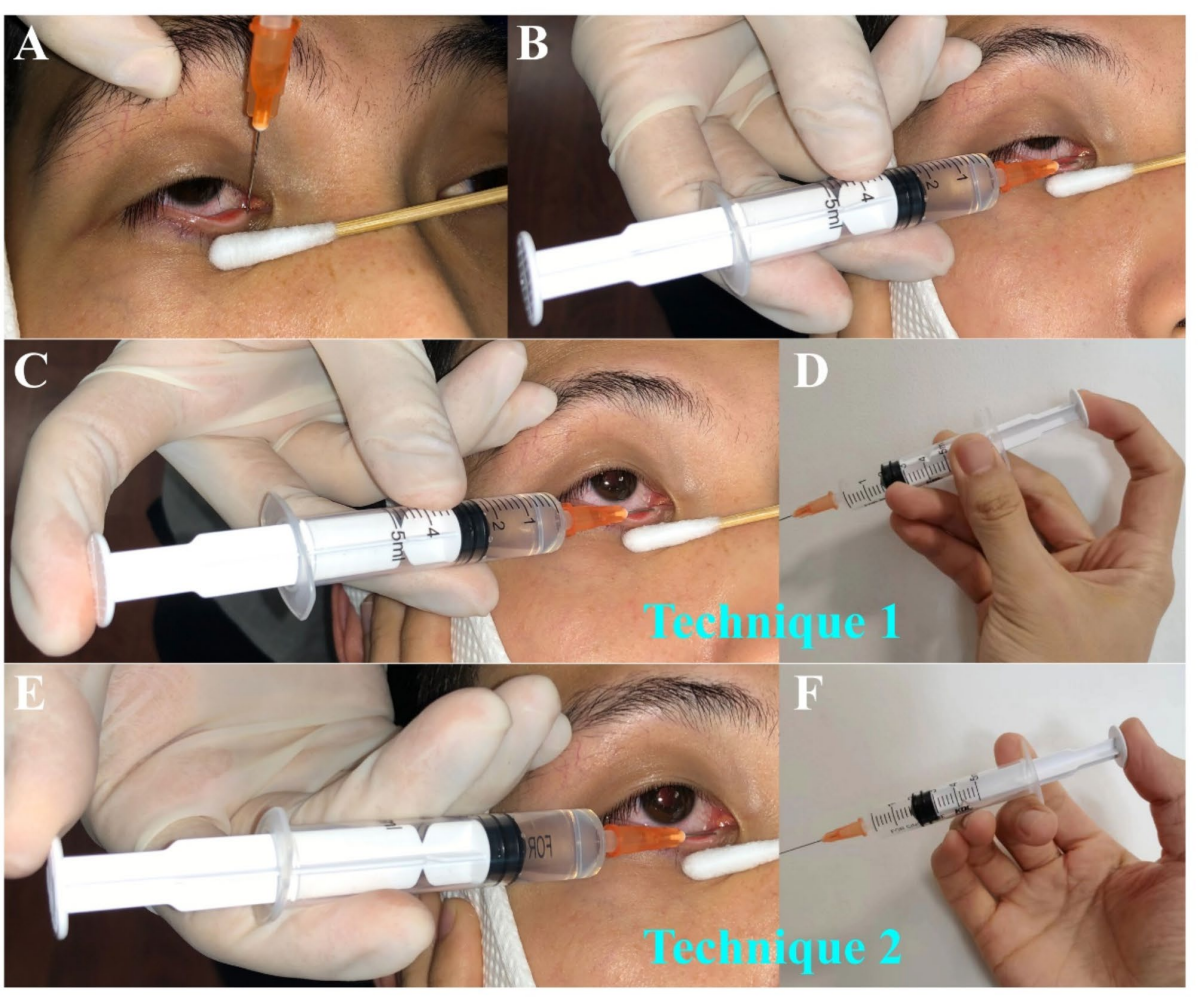
Lacrimal Irrigation Process: **A**. Vertically insert syringe into the lower punctum by approximately 1.5–2 mm. **B**. Adjust syringe for horizontal injection. **C-D**. Maintain a firm grip on the syringe barrel with the thumb and middle finger, while the index finger charges the syringe plunger. **E-F**. Create a crossed fixation of the barrel between the index and middle fingers, and reposition the thumb to the syringe plunger

For the lower lacrimal puncta, the procedure started by instructing the patient to look upward. The operator gently pulled the lower eyelid outward and downward, exposing the lower punctum upward. Holding the syringe with the thumb, index finger, and middle finger, the operator inserted the cannula vertically into the lower punctum by about 1.5–2 mm. A distinct sense of penetration was felt (Fig. 2A). Then, the syringe was adjusted for a horizontal injection (Fig. 2B). As the cannula approached the lacrimal sac, the operator tilted the syringe downward by approximately 15° while steadily advancing upward. When the cannula tip encountered a hard stop as it passed beyond the common canaliculus and reached the anterior lacrimal crest, it was retracted by 2–3 mm. Subsequently, the operator adjusted their hand position to prepare for the injection of saline solution.

There are two specific techniques outlined below:

##### Technique 1

Maintain a firm grip on the syringe barrel using the thumb and middle finger while releasing the index finger to transfer it to the syringe plunger. Push the plunger to perform lacrimal irrigation (Fig. 2C-D).

##### Technique 2

Maintain a secure hold on the syringe barrel with the thumb and index finger. Transfer the middle finger to the opposite side of the syringe, creating a crossed fixation of the barrel between the index and middle fingers. Once the syringe is stabilized, reposition the thumb to the syringe plunger and push the plunger to perform lacrimal irrigation (Fig. 2E-F).

During the lacrimal irrigation process, fluid is gently injected while carefully monitoring for fluid reflux. The patient is asked if they perceive any fluid entering the throat. Following the completion of the procedure, Tobramycin eye drops are administered, and the results of lacrimal irrigation are documented.

During the process, the following findings were noted:

- Some participants initially struggled with the correct insertion angle, causing patient discomfort. Based on operator feedback, adjustments were made to refine the technique and improve procedural accuracy.
- The presence of resistance or a “hard stop” when the cannula reached the anterior lacrimal crest was critical for understanding proper insertion depth.
- Participants’ skill levels varied significantly, with more proficient operators exhibiting better control over the syringe and less discomfort reported by patients.

## Design rationale and key features of the haptic-visual integrated self-serve training model

To enhance the design of the training model, we conducted preliminary experiments involving various finger webs on the left-hand glove and even tested placing gloves over disposable paper cups. Ultimately, we determined that the finger web positioned between the index finger and thumb provided the optimal design for the training model. This choice was based on several key considerations:

### Tactile sensitivity

The skin between the index finger and thumb is highly sensitive, providing realistic haptic feedback, enabling operators to acutely perceive the penetration sensation during cannula insertion, as well as the trajectory and depth variations of the cannula within the designated path. This heightened sensitivity facilitates the immediate detection and correction of improper techniques during training.

### Operational space

This area provides sufficient space for cannula manipulation while maintaining a relatively stable support structure, owing to the stronger and more coordinated force generated by the combination of the thumb and index finger compared to other digits. This stability allows trainees to practice precise movements and sustain training for longer durations.

Supplies for the preparation include a marker pen, a 5-mL syringe, a blunt-tipped 23-G lacrimal cannula, saline solution, disposable light-colored rubber gloves, and a ruler.

For the training model design, follow these steps:

### Step 1

Simulate tense skin with the glove.

Put on a disposable light-colored rubber glove and spread open your index finger and thumb to create tension in the glove over the finger web.

### Step 2

Create a simulated punctum.

**Fig. 3:**
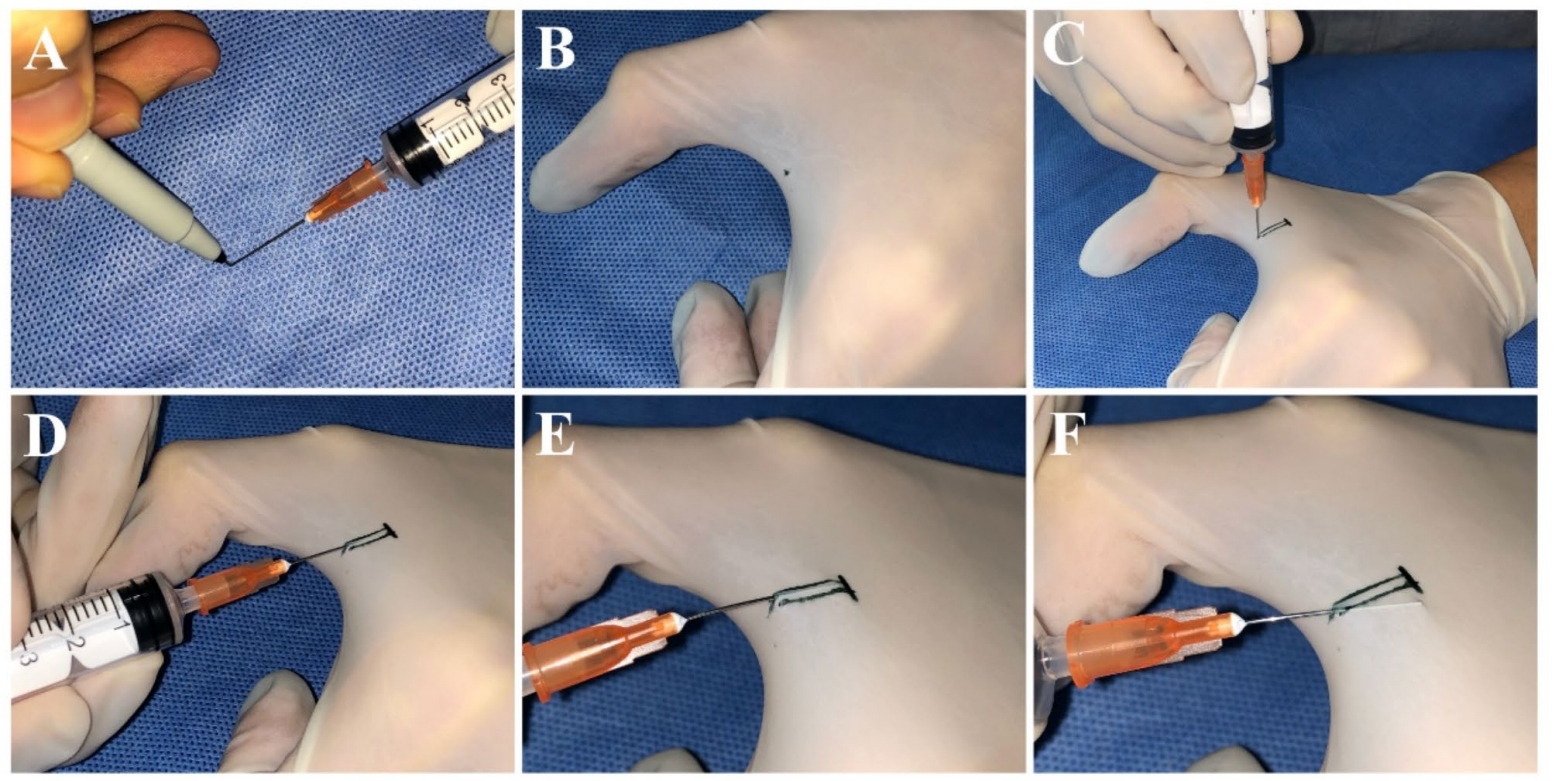
Model training key points: **A**. Use a marker to apply ink to the blunt-tipped lacrimal cannula. **B**. Create a marked punctum resembling the lacrimal punctum’s shape. **C**. Draw two parallel lines, 10–12 mm long, spaced 2–3 mm apart to mimic the lacrimal canaliculus. **D**. Gently advance the cannula 8–10 mm along the simulated lacrimal duct, ensuring cannula visibility through the latex glove. **E**. Noticing a distinct cannula shape indicates excessive lifting. **F**. Avoid tilting the cannula beyond the marked lines

Using a marker pen, apply ink to the tip of the blunt-tipped lacrimal cannula (Fig. 3A). Position the marked cannula at the central edge between the index finger and thumb, piercing the glove to create a marked punctum that mimics the shape of the lacrimal punctum. This punctum will be used for subsequent lacrimal duct irrigation training (Fig. 3B).

### Step 3

Create a simulated lacrimal canaliculus and the bony wall.

Using a marker pen, draw two parallel lines perpendicular to the finger web, approximately 10–12 mm in length and spaced 2–3 mm apart, to simulate the lacrimal canaliculus. At the far end of the lines from the puncture site, draw a vertical line to indicate the position where the distal end of the lacrimal sac contacts the anterior lacrimal crest (Fig. 3C). This marks the maximum insertion depth that the cannula should reach during lacrimal irrigation training. Do not exceed this marked line.

## Key usage points

### Vertical insertion of lacrimal Punctum

During training, hold a syringe filled with 3 ml of air and align it with the marked simulated lacrimal punctum. Insert the cannula vertically to a depth of 2 mm, feeling a similar empty penetration sensation as with a real punctum (Fig. 3C). Adjust the depth based on tactile feedback on the finger web. Avoid inserting the cannula too deeply, as it may cause sharp pain.

### Slow and horizontal insertion within marked line

After vertical insertion, rotate the cannula horizontally towards the finger web and gradually advance it along the marked simulated lacrimal duct by 8–10 mm. Ensure the cannula remains within the marked line and avoid tilting it (Fig. 3F), which indicates an incorrect insertion angle. The shape of the cannula should be visible through the latex glove, indicating the appropriate depth of insertion (Fig. 3D). Observing a noticeable cannula shape suggests excessive lifting, while causing discomfort on the finger indicates excessive pressing down (Fig. 3E).

### Switch hand gestures and simulate injection

When the cannula’s tip reaches the endpoint marked line, retract it by 2–3 mm, and then switch hand gestures to inject the air within the syringe, simulating the injection of a saline solution. After completing the injection, slowly withdraw the cannula.

### Enhance training based on haptic feedback

Adjust the training based on the tactile feedback of the skin. Consistent and effective training sessions will improve proficiency and stability in lacrimal irrigation, allowing operators to master the skill and prepare for real clinical practice.

### Statistical analysis

Statistical analysis utilized SPSS Statistical Software, V.20 (IBM, SPSS). Continuous variables are presented as mean ± SD or median (range), determined by the Shapiro-Wilk normality test. Student’s t-test compared differences between two data groups. Nonparametric significance tests, including the Mann-Whitney U test, chi-square test, or Fisher’s exact test, were employed as appropriate. Statistical significance was set at *p* < 0.05. All participants provided complete datasets. No missing data required imputation or exclusion.

## Results

### Participants characteristics

All 92 participants completed the study, with 47 in Group A and 45 in Group B. The cohort consisted of 61 females (66.3%) and 31 males (33.7%), with a mean age of 24.30 ± 2.45 years (range: 21–33). Among the participants, 21 had prior experience with lacrimal irrigation, indicating a certain level of familiarity, while 71 had no prior experience in this area. Baseline characteristics, including age, gender distribution, and prior experience with lacrimal irrigation, showed no significant differences between Group A and Group B (*p* > 0.05), confirming that the groups were well-balanced at baseline (Table 1).

**Table 1:** Baseline characteristics of the participants in group A and group B

	Group A (*n* = 47)	Group B(*n* = 14)	*p* value
Gender			
Male	15	16	0.826
Female	32	29	
Age	24.64 ± 2.633	23.96 ± 2.225	0.183
Prior experience			
Yes	14	7	0.104
No	33	38	

### Assessing trainees’ skill proficiency after training

After observing demonstrations of two different lacrimal irrigation techniques, trainees from group A independently selected one technique to practice. In group A, 70.2% of trainees (33/47) chose technique one (group A1), while 29.8% of trainees (14/47) chose technique two (group A2). There were no significant differences in terms of gender, age and baseline skill ability between groups A1 and A2 (*p* > 0.05). Trainees completed an average of 2.98 ± 1.54 (range: 1–10) practice sessions for lacrimal irrigation, and there was no statistically significant difference between groups A1 and A2 (*p* > 0.05). Based on the self-assessed mastery level in the post-training questionnaire, 35 trainees (74.4%) reported partial mastery of the skill, 9 trainees (19.1%) reported basic mastery, and 3 trainees (6.4%) reported proficiency. There was no statistically significant difference between groups A1 and A2 in terms of self-assessed mastery level (*p* > 0.05) (Table 2).

**Table 2:** Detailed scores of lacrimal irrigation performance of the participants in group A1 and A2

	Group A1(*n* = 33)	Group A2(*n* = 14)	*p* value
Gender			
Male	8	7	0.083
Female	25	7	
Age	24.39 ± 2.549	25.21 ± 2.833	0.249
Baseline of skills ability			
Novices	25	8	0.354
Entry-level individual	8	6	
Practice times	3.12 ± 0.30	2.64 ± 0.25	0.335
Mastery level			
Partial mastery	27	8	0.159
Basic proficiency	5	4	
Advanced proficiency	1	2	

### The analysis of lacrimal irrigation techniques

Based on the survey results (Table 3), 66.7% (22/33) of participants in group A1 found technique one easy to master. Similarly, 85.7% (12/14) of participants in group A2 found technique two easy to master. There was no significant difference in the perceived difficulty of mastering these two techniques (*p* > 0.05). The handling and stability survey for both techniques were also evaluated, and no significant difference was found between group A1 and A2 (*p* > 0.05). However, technique two was found to be easier for performing the injection compared to technique one (*p* < 0.05). Additionally, participants rated the comfort of the lacrimal irrigation procedure using the Numeric Rating Scale based on patient complaints and facial expressions. The comfort rating for technique one was 2.06 ± 1.73, while for technique two, it was 1.57 ± 1.79. There was no significant difference in comfort ratings between the two groups (*p* > 0.05). Furthermore, the Skill assessment scores were 81.63 ± 7.72 in group A1 and 84.22 ± 8.85 in group A2. There was also no significant difference in Skill assessment scores (*p* > 0.05).

**Table 3:** The analysis of lacrimal irrigation techniques

	Group A1(*n* = 33)	Group A2(*n* = 14)	*p* value
Mastery difficulty			
Easy	22	12	0.328
Difficult	11	2	
Stability			
Stable	15	8	0.464
Unstable	18	6	
Perform injection			
Easy	19	13	0.018
Difficult	14	1	
Comfort rating	2.06 ± 1.73	1.57 ± 1.79	0.385
Skill assessment scores	81.63 ± 7.72	84.22 ± 8.85	0.320

### Factors influencing lacrimal irrigation skill mastery: insights from Post-Training questionnaires

**Fig. 4:**
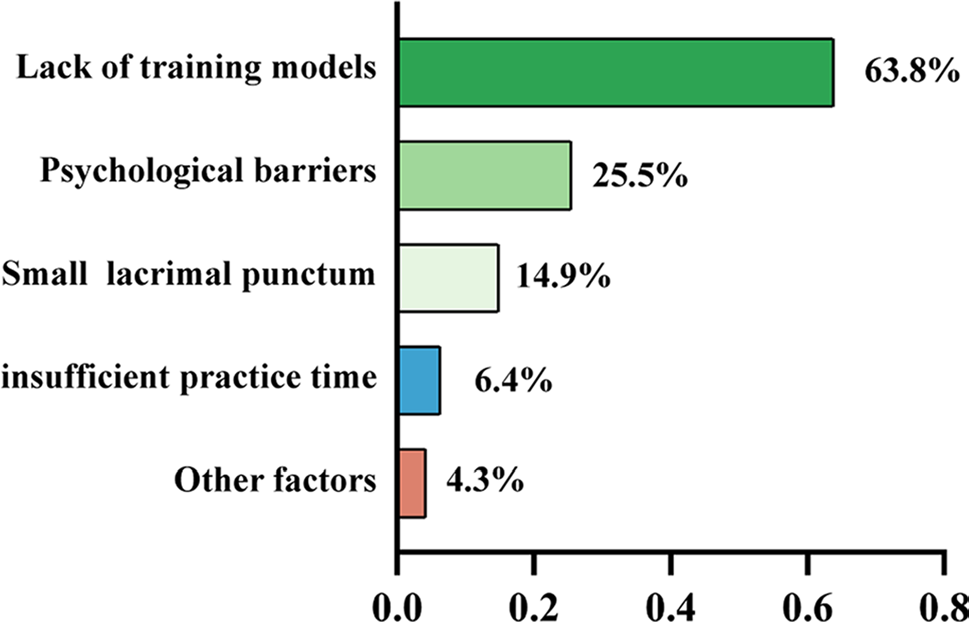
Key challenges hindering lacrimal irrigation skill acquisition

In order to further explore the factors affecting lacrimal irrigation skill mastery, we analyzed data from post-training questionnaires. The analysis revealed that the predominant factor was the absence of appropriate training models, as noted by 63.8% (30 out of 47) participants. A further 25.5% (12 out of 47) cited psychological barriers encountered during the procedure. Other noted challenges included the punctal stenosis, as reported by 14.9% (7 out of 47), and a lack of adequate practice time, mentioned by 6.4% (3 out of 47). Additionally, the limited access to practicing with live patients was recognized by 4.3% (2 out of 47) as an impediment to skill acquisition. (Fig. 4)

### Effectiveness of the lacrimal training model

Based on feedback from Group A, we developed a haptic-visual integrated self-serve training model. Participants were allowed to select their preferred lacrimal irrigation technique during training, as preliminary analysis revealed no significant difference in skill acquisition efficiency between the two techniques (*p* > 0.05). Evaluation of the training model demonstrated that Group B1 (model users) achieved significantly higher confidence scores compared to Group B2 (non-users) (*p* < 0.05). While Group B1 showed a trend toward improved skill assessment scores, this difference did not reach statistical significance (*p* = 0.167). Notably, 85% of Group B1 participants rated the training model as highly effective for skill development (Table 4).

**Table 4:** The analysis of efficacy of the training model

	Group B1(*n* = 20)	Group B2(*n* = 19)	*p* value
Skill assessment scores	80.741 ± 8.321	76.817 ± 9.075	0.167
Confidence score	6.850 ± 1.725	5.526 ± 1.954	0.031
Skill-enhancing or not?			
Yes	17(85%)	/	
No	3(15%)	/	

## Discussion

This study demonstrates that both lacrimal irrigation techniques can be effectively mastered by novice ophthalmology residents, with no significant difference in skill acquisition efficiency (*p* > 0.05). Although Technique 1 was preferred by 72% of participants due to its perceived ease of use, Technique 2 demonstrated superior performance in pressurized irrigation scenarios, a critical requirement for diagnosing partial obstructions [2, 13]. This aligns with our skill assessment data, which revealed comparable performance between the two techniques, suggesting that user preference may be influenced by ergonomic factors rather than technical superiority.

Teaching invasive skills, such as lacrimal irrigation, to junior physicians is a resource-intensive process fraught with challenges. The delicate nature of the lacrimal drainage system results in low fault tolerance during procedures, while the risk of ocular injury induces psychological apprehension among trainees [9, 15]. Furthermore, the absence of standardized training models raises ethical concerns, particularly when practicing on live patients. These challenges are exacerbated when operators lack sufficient proficiency, underscoring the need for effective training solutions [16–18].

The self-serve training model developed in this study significantly enhanced operator confidence (Group B1: 6.9 ± 1.7 vs. B2: 5.5 ± 2.0, *p* = 0.031), supporting the evidence that procedural confidence is a crucial mediator in the development of technical competence for invasive skills. Higher levels of confidence are closely linked to improved surgical performance and increased motivation, which includes greater persistence and effort during skill acquisition [19, 20]. This finding is consistent with broader evidence in surgical education, where confidence, when coupled with deliberate practice, positively correlates with objective measures of procedural skill [21]. Moreover, the alignment between confidence and competence is essential for ensuring safe clinical practice, as both overconfidence and underconfidence can lead to clinical errors. In this context, the Confidence-Competence Ratio (CCR) serves as a valuable framework to guide learners toward achieving a balanced skillset [22].

While 85% of Group B1 participants perceived the model as effective, its limited impact on objective skill scores (B1: 80.7 ± 8.3 vs. B2: 76.8 ± 9.1, *p* = 0.167) highlights two key insights. First, the assessment scope mismatch between the model’s focus on procedural execution and the comprehensive evaluation rubric—which included pre-procedural, procedural, and post-procedural components—may have diluted its measurable impact [23]. Second, the temporal dynamics of skill acquisition suggest that confidence gains often precede measurable improvements, particularly in early-stage learners [24]. While our 8-hour intervention effectively boosted confidence, it may have been insufficient to surpass the “novice plateau” effect. The non-significant trend toward improved skill scores (*p* = 0.167) implies that extended practice with the model could translate confidence gains into statistically significant competency improvements—a hypothesis warranting further investigation.

Despite its diagnostic limitations—such as the inability to distinguish between nasolacrimal duct stenosis and functional delay [13], or identify pre-sac etiologies in cases of epiphora [12, 25], —lacrimal irrigation remains indispensable for preoperative assessment, particularly in reducing the risk of post-surgical endophthalmitis during intraocular surgery [5, 26–28]. In this context, non-invasive alternatives like the regurgitation on pressure over the lacrimal sac (ROPLAS) test have been proposed as complementary screening tools [1, 29]. ROPLAS, a simple outpatient procedure with minimal patient discomfort, is particularly valuable in resource-limited settings and has been shown to be effective for initial screening of nasolacrimal duct obstruction (NLDO) [30]. However, its limitations—including low sensitivity and predictive value—highlight the need for confirmatory testing with lacrimal irrigation in ambiguous cases [31]. Thus, while ROPLAS offers a quick, patient-friendly screening option, lacrimal irrigation remains indispensable for comprehensive preoperative assessment [2].

Given the critical role of lacrimal irrigation in diagnosing lacrimal diseases, mastering this skill is essential for ophthalmologists [28]. Our self-serve training model developed in this study addresses three key barriers to proficiency: (1) improved fault tolerance through simulated tactile feedback, allowing trainees to practice and correct errors in a low-risk environment, (2) psychological safety by eliminating risks associated with live patient procedures, and (3) standardization by providing reproducible practice conditions absent in clinical apprenticeships.

However, the model has notable limitations. First, it cannot simulate two-handed techniques requiring simultaneous eyelid manipulation and cannula advancement, a fundamental clinical skill that needs future integration. Second, its ecological validity is constrained by the absence of physiological responses, such as mucosal resistance variations, which influence real-world irrigation dynamics.

To address these limitations, we propose three future directions: (1) enhanced simulation through the development of specialized training gloves with force-sensitive membranes to mimic mucosal resistance gradients, (2) longitudinal validation studies with a larger sample size to assess skill retention and clinical error rates over a 6-month period, and (3) competency mapping using motion analytics (e.g., cannula path deviation metrics) to complement subjective assessments. Looking ahead, technologies like virtual reality (VR) and artificial intelligence (AI) could enhance lacrimal irrigation training by providing immersive simulations and real-time feedback, further improving skill acquisition and standardization.

## Conclusion

This study demonstrates that both lacrimal irrigation techniques are equally accessible to novices, with Technique 2 offering distinct advantages in pressurized irrigation scenarios. The self-serve training model significantly enhances procedural confidence—a vital precursor to clinical competence—though technical proficiency requires extended deliberate practice beyond initial confidence gains. Despite limitations in short-term evaluation and partial anatomical fidelity, the model provides a scalable, risk-free platform for skill acquisition, addressing critical ethical and resource constraints. Future advancements, including enhanced simulation techniques, physiological feedback systems, and refined evaluation metrics, hold promise for bridging the gap between simulated training and clinical performance.

## Electronic supplementary material

Below is the link to the electronic supplementary material.

Supplementary Material 1

Supplementary Material 2

## Data Availability

The data presented in this study are available from the corresponding author on reasonable request.
