# Supplementary material for: A comparative study of irrigation techniques and the development of a self-serve training model for ophthalmology residents
Source: BMC Med Educ. 2025 Mar 6;25:344. doi: 10.1186/s12909-025-06889-2 (PMC11884064; doi:10.1186/s12909-025-06889-2)
Supplement: Supplementary file 1 — Supplementary Material 1 [file 12909_2025_6889_MOESM1_ESM.docx]

**Supplementary Table 1.** **Post-training questionnaire for Group A1 and A2**

| Question | | Answer |
| --- | --- | --- |
| Have you had any prior experience with lacrimal syringing procedures before commencing this training session? | | - Novices - Entry-level individual   ( Entry-level skill ability was defined as the ability to independently and successfully perform lacrimal irrigation with a history of ≤10 prior attempts.) |
| Which lacrimal syringing techniques do you prefer to employ in your practice | | - Technique 1 - Technique 2 |
| How many lacrimal syringing procedures did you perform during this training? | | _____times |
| What is your evaluation of the lacrimal syringing techniques that you have chosen to employ in your practice? | Mastery difficulty | - Easy - Relatively difficult |
|  | Stability | - Stable - Relatively unstable |
|  | Perform injection | - Easy - Relatively difficult |
| How do you assess patients’ comfort levels during the lacrimal syringing procedures you perform? | | From 1-10; 0= no pain, 10= excruciating pain.  1 2 3 4 5 6 7 8 9 10 |
| What is your assessment of your mastery level in lacrimal syringing after completing the training? | | - Partial mastery - Basic proficiency - Advanced proficiency |
| What do you consider to be the significant factor hindering your mastery of lacrimal syringing technique? | | - Lack of lacrimal syringing practice models; - Punctal stenosis, making practice difficult; - Inability to overcome psychological barriers during lacrimal syringing procedures; - Not having enough time to practice the procedure; - Other___________ |
