# Supplementary material for: A comparative study of irrigation techniques and the development of a self-serve training model for ophthalmology residents
Source: BMC Med Educ. 2025 Mar 6;25:344. doi: 10.1186/s12909-025-06889-2 (PMC11884064; doi:10.1186/s12909-025-06889-2)
Supplement: Supplementary file 2 — Supplementary Material 2 [file 12909_2025_6889_MOESM2_ESM.docx]

**Supplementary Table 2.** **Post-training questionnaire for Group B1 and B2**

| Question | | Answer | |
| --- | --- | --- | --- |
| What is your confidence level with the following after completing the training? | | From 1-10; 1=least confidence, 10= highest confidence. 1 2 3 4 5 6 7 8 9 10 | |
| Do you apply the self-serve training model? | - No; |  | |
|  | - Yes; | Skill-enhancing or not? | - Yes; - No; |
